# Supplementary material for: The Streptococcus pyogenes mannose phosphotransferase system (Man-PTS) influences antimicrobial activity and niche-specific nasopharyngeal infection
Source: J Bacteriol. 2025 Mar 26;207(4):e00492-24. doi: 10.1128/jb.00492-24 (PMC12004959; doi:10.1128/jb.00492-24)
Supplement: Supplemental figures and tables — Fig. S1 to S3; Tables S1 to S6. [file jb.00492-24-s0001.pdf]

SUPPLEMENTARY MATERIAL

The *Streptococcus pyogenes* mannose phosphotransferase system (Man-PTS) influences antimicrobial activity and niche-specific nasopharyngeal infection

Amanda C. Marple, Blake A. Shannon, Aanchal Rishi, Lana Estafanos, Brent D. Armstrong, Veronica Guariglia Oropeza, Stephen W. Tuffs*, John K. McCormick

Department of Microbiology and Immunology, University of Western Ontario, London, ON, Canada.

Address correspondence to John K. McCormick, john.mccormick@uwo.ca

Tel. (+1) 519-661-3309

Fax (+1) 519-661-3499

* Present address: Stephen W. Tuffs, Department of Biochemistry and Microbiology, University of Victoria, Victoria, British Columbia, Canada

Short title: *S. pyogenes* sugar metabolism and niche specificity

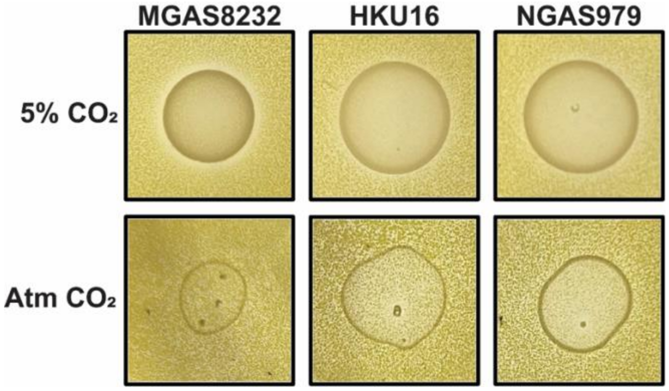

Figure S1. *S. pyogenes* HKU16 and NGAS979 produce an antimicrobial phenotype similar to *S. pyogenes* MGAS8232. *S. pyogenes* strains MGAS8232, HKU16, and NGAS979 were spotted on GalM17 in 5% CO_2 and atmospheric levels of CO_2. After 24 hours of incubation at 37°C, soft agar with *M. luteus* was overlayed and incubated for another 24 hours at the same temperature. Zones of inhibition on the plates represent antimicrobial activity from the spotted bacterium.

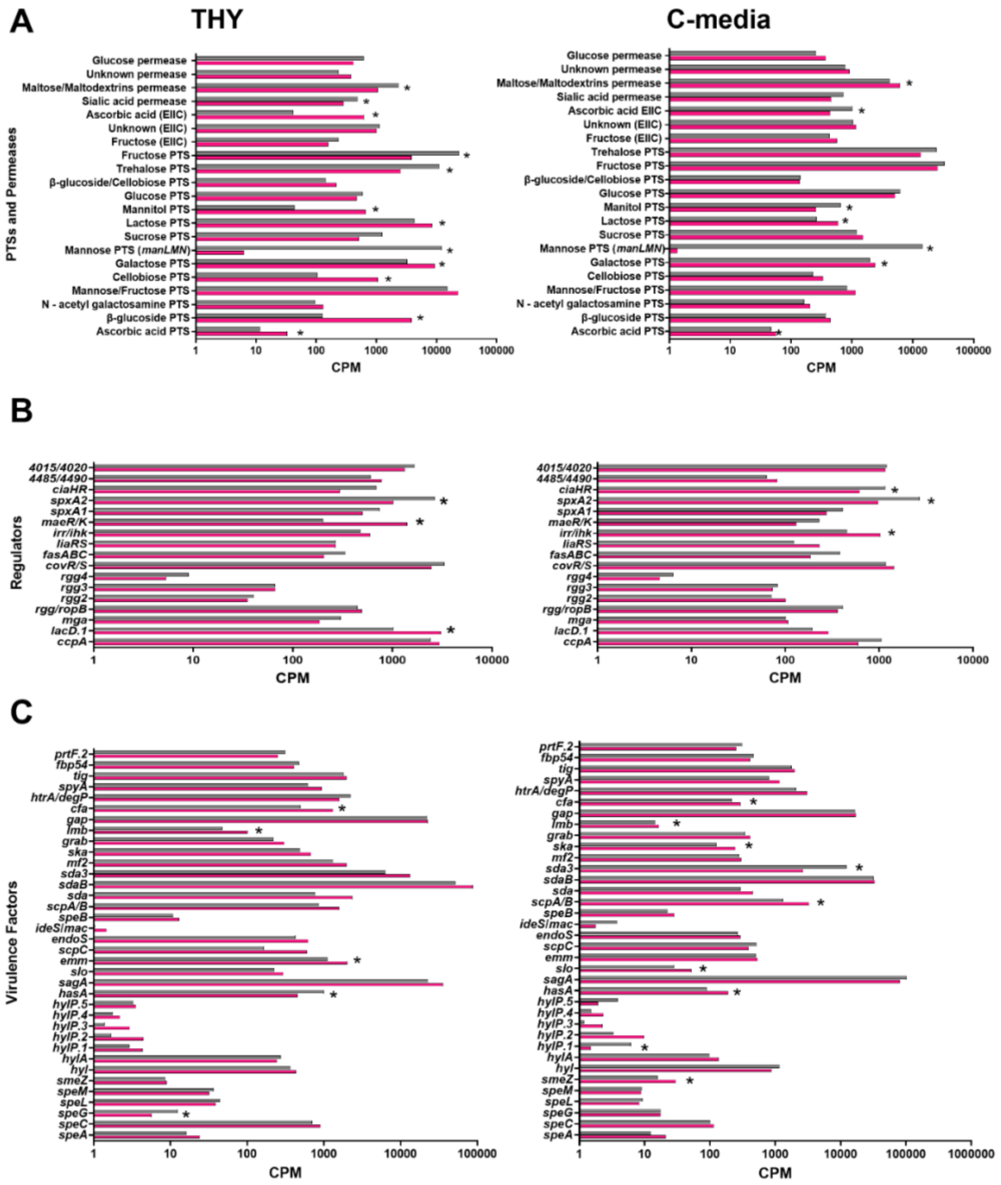

FIG S2. Transcripts of sugar PTSs and permeases, regulators, and virulence factors in wildtype *S. pyogenes* MGAS8232 and the Δ*manLMN* strain when grown in high and low glucose environments. *S. pyogenes* MGAS8232 and Δ*manLMN* were grown in THY (n=3; OD_{600} of 0.7 - 0.8) or C-media (n=3; OD_{600} of 0.6-0.7). Samples with RNA integrity numbers (RIN) of ≥ 7 were subjected to RNA-seq. The following represents the transcripts (counts per million (CPM)) from genes related to each (**A**) sugar PTS and permease, (**B**) virulence regulators, and (**C**) virulence factors that are encoded in *S. pyogenes* MGAS8232. All virulence factors were identified via the virulence factor database (VFDB: Virulence Factors of Bacterial Pathogens (mgc.ac.cn)). PTSs, permeases, and virulence regulators that consist of more than one gene are represented by the sum of their transcripts. The transcripts from wildtype MGAS8232 and the Δ*manLMN* strain are represented in grey and pink bars, respectively. * Represents at least one gene that had $p \leq 0.05$.

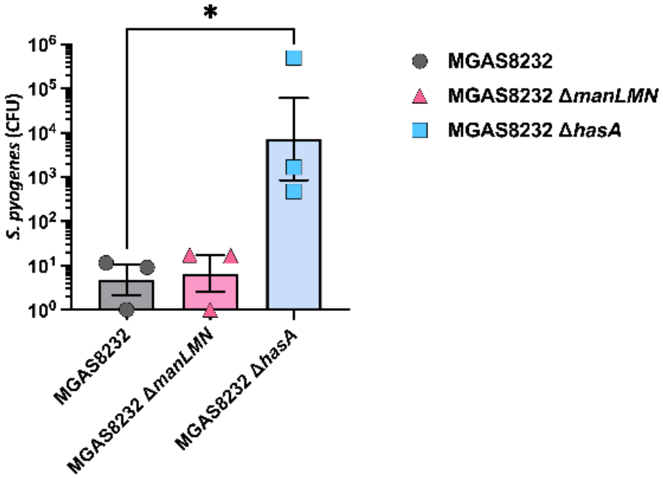

FIG S3. The reduction of capsule in *S. pyogenes* Δ*manLMN* does not cause increased invasion in Detroit-562 cells. Internalization assay of wild-type *S. pyogenes* MGAS8232 via a modified classical gentamycin assay comparing the Man-PTS (Δ*manLMN*) and hyaluronic acid capsule (Δ*hasA*) deficient mutants in Detroit 562 cells. D562 cells were cultured with *S. pyogenes* (MOI 100) for 2 hours at 37°C with 5% CO_2, then supplemented with 100µg mL^{-1} of gentamycin for 1.5 hours. Bars represent geometric mean, and each dot is a biological replicate. *, $p \leq 0.1$ via One-Way ANOVA.

Table S1. Single nucleotide polymorphisms and genetic alterations in the Spb clean deletion mutants

	Single Nucleotide Polymorphisms							
	Position	Type	Ref	Alt	AA change	Effect	Gene	Locus tag
ΔspbJK	354777	del	CT	C	Ser27fs	frameshift	HlyC/CorC family transporter	SPYM18_RS01955
	1873136	del	AT	A	Ile140fs	frameshift	insulinase family protein	SPYM18_RS09550
ΔspbMN	96100	snp	T	C	Pro619Pro	synonymous	*rpoB*, DNA-directed RNA polymerase subunit beta	SPYM18_RS00630
	1450278	snp	T	C	Met410Val	missense	NCS2 family permease	SPYM18_RS07500
	1753971	snp	T	C	Glu47Gly	missense	streptopain	SPYM18_RS09005
ΔspbJKMN	96100	snp	T	C	Pro619Pro	synonymous	*rpoB*, DNA-directed RNA polymerase subunit beta	SPYM18_RS00630
	987920	snp	G	T	Ala499Glu	missense	ABC transporter ATP-binding protein	SPYM18_RS05100
	1140999	snp	G	A	Ala83Val	missense	LPXTG cell wall anchor domain-containing protein	SPYM18_RS05925
	1421847	snp	T	C	Thr225Ala	missense	Na/Pi cotransporter family protein	SPYM18_RS07330
	1450278	snp	T	C	Met410Val	missense	NCS2 family permease	SPYM18_RS07500
	1496942	snp	G	A	Trp117*	stop gained	*manN*, PTS mannose/fructose /sorbose transporter family subunit IID	SPYM18_RS07830
	1498186	snp	G	T	N/A	N/A	N/A	N/A
	1753971	snp	T	C	Glu47Gly	missense	streptopain	SPYM18_RS09005
	1763350	snp	G	A	N/A	N/A	N/A	N/A
	1765535	del	CT	C	N/A	N/A	N/A	N/A
	1873136	del	AT	A	Ile140fs	frameshift	insulinase family protein	SPYM18_RS09550

Note: AA=amino acid, del=deletion, fs=frameshift, snp=single nucleotide polymorphism, *=stop codon. Available at NCBI Bioproject PRJNA1176168.

Table S2. Single nucleotide polymorphisms and genetic alterations in the transposon mutants

	Single Nucleotide Polymorphisms							
	**Position**	**Type**	**Ref**	**Alt**	**AA change**	**Effect**	**Gene**	**Locus tag**
***manN*::tn (07830)**	229153	snp	C	T	Ser828Phe	missense	SEC10/PgrA surface exclusion domain-containing protein	SPYM18_RS02175
	1364445	snp	G	A	Thr553Ile	missense	primosomal protein N'	SPYM18_RS07080
	1675147	snp	G	A	Ala413Val	missense	*pnp*, polyribonucleotide nucleotidyltransferase	SPYM18_RS08685
	1873457	del	AT	A	Ile33fs	frameshift	insulinase family protein	SPYM18_RS09550
***galC*::tn (07380)**	229153	snp	C	T	Ser828Phe	missense	SEC10/PgrA surface exclusion domain-containing protein	SPYM18_RS02175
	1364445	snp	G	A	Thr553Ile	missense	primosomal protein N'	SPYM18_RS07080
	1390878	snp	G	A	Ala134Val	missense	amino acid ABC transporter permease	SPYM18_RS07200
	1675169	snp	G	A	His406Tyr	missense	*pnp*, polyribonucleotide nucleotidyltransferase	SPYM18_RS08685
	1873457	del	AT	A	Ile33fs	frameshift	insulinase family protein	SPYM18_RS09550
***lacA*::tn (08595)**	229153	snp	C	T	Ser828Phe	missense	SEC10/PgrA surface exclusion domain-containing protein	SPYM18_RS02175
	1364445	snp	G	A	Thr553Ile	missense	primosomal protein N'	SPYM18_RS07080
	1420865	snp	G	A	N/A	N/A	N/A	N/A
	1873457	del	AT	A	Ile33fs	frameshift	insulinase family protein	SPYM18_RS09550

Note: AA=amino acid, snp=single nucleotide polymorphism, del=deletion, fs=frameshift, N/A=not available. Available at NCBI Bioproject PRJNA1176168.

Table S3. Single nucleotide polymorphisms and genetic alterations in the Man-PTS deficient strain.

	Single Nucleotide Polymorphisms							
	Position	Type	Ref	Alt	AA change	Effect	Gene	Locus tag
ΔmanLMN	401924	del	GA	G	Lys110fs	frameshift	metal ABC transporter ATP-binding protein	SPYM18_RS02175
	1263863	snp	G	A	N/A	N/A	N/A	N/A

Note: AA=amino acid, del=deletion, fs=frameshift, snp=single nucleotide polymorphism. Available at NCBI Bioproject PRJNA1176168.

Table S4. Differential expression analyses of *S. pyogenes* MGAS82323 Δ*manLMN* and wildtype *S. pyogenes* MGAS8232 at late exponential phase in THY.

Locus tag	Gene	Description	$\log_2$ (fold change)	$-\log_{10}$ (*p* value)
SPYM18_RS00940		PTS sugar transporter subunit IIC	1.477	2.298
SPYM18_RS00945		PTS sugar transporter subunit IIB	1.525	2.330
SPYM18_RS00950		PTS sugar transporter subunit IIA	1.545	2.324
SPYM18_RS00955		3-keto-L-gulonate-6-phosphate decarboxylase UlaD	1.755	2.211
SPYM18_RS00960		L-ribulose-5-phosphate 3-epimerase	1.863	2.110
SPYM18_RS01055		helix-turn-helix domain-containing protein	2.088	2.060
SPYM18_RS01105		carbonic anhydrase	-1.662	2.019
SPYM18_RS01120		sensor histidine kinase	-1.183	2.046
SPYM18_RS01275		amino acid ABC transporter ATP-binding protein	-0.897	2.318
SPYM18_RS02070	*scpC*	CXC chemokine-degrading serine protease SpyCEP	1.845	2.137
SPYM18_RS02085	*nrdF*	class 1b ribonucleoside-diphosphate reductase subunit beta	2.542	4.563
SPYM18_RS02090	*nrdI*	class Ib ribonucleoside-diphosphate reductase assembly flavoprotein NrdI	2.347	3.732
SPYM18_RS02535	*dhaL*	dihydroxyacetone kinase subunit DhaL	1.601	2.273
SPYM18_RS02540	*dhaM*	dihydroxyacetone kinase phosphoryl donor subunit DhaM	1.401	2.057
SPYM18_RS02545		MIP/aquaporin family protein	1.514	2.008
SPYM18_RS02750		PRD domain-containing protein	5.536	7.828
SPYM18_RS02755		PTS beta-glucoside transporter subunit IIBCA	4.926	6.456
SPYM18_RS02760		glycoside hydrolase family 1 protein	4.901	6.703
SPYM18_RS02765		TIGR03943 family protein	-0.996	2.599
SPYM18_RS02770		permease	-1.146	2.188
SPYM18_RS02780		Tex family protein	-1.140	3.076
SPYM18_RS02820		peptidase U32 family protein	-0.992	2.187
SPYM18_RS03235		terminase large subunit	1.238	2.496
SPYM18_RS03250		Clp protease ClpP	1.977	2.560
SPYM18_RS03255		phage major capsid protein	1.932	2.801
SPYM18_RS03365		zinc ABC transporter substrate-binding protein AdcA	1.073	2.124
SPYM18_RS03515	*atpB*	F0F1 ATP synthase subunit A	-0.948	2.229
SPYM18_RS03525		F0F1 ATP synthase subunit delta	-1.197	2.418
SPYM18_RS03895		cation diffusion facilitator family transporter	-2.331	3.910
SPYM18_RS03935	*pfkB*	1-phosphofructokinase	-1.778	2.570
SPYM18_RS03940		fructose-specific PTS transporter subunit EIIC	-2.641	2.960
SPYM18_RS03945		glycoside hydrolase family 73 protein	-2.056	4.519
SPYM18_RS04180		branched-chain amino acid aminotransferase	-1.049	2.693
SPYM18_RS04185		DUF2969 domain-containing protein	-2.343	2.246
SPYM18_RS04255	*recJ*	single-stranded-DNA-specific exonuclease RecJ	-0.923	2.038
SPYM18_RS04325		tryptophan ABC transporter substrate-binding protein	-1.016	2.294

SPYM18_RS04330		ABC transporter permease	-1.256	3.002
SPYM18_RS04605		response regulator	2.758	4.980
SPYM18_RS04610		sensor histidine kinase	2.821	6.328
SPYM18_RS04615		2-hydroxycarboxylate transporter family protein	5.712	7.418
SPYM18_RS04620		NADP-dependent malic enzyme	5.791	8.475
SPYM18_RS04630	*aphA*	acid phosphatase AphA	1.598	3.397
SPYM18_RS04725	*guaC*	GMP reductase	-1.696	2.422
SPYM18_RS04775		serine hydroxymethyltransferase	-1.231	2.043
SPYM18_RS04795		ABC transporter ATP-binding protein	-1.094	2.455
SPYM18_RS04840		DUF1836 domain-containing protein	1.866	2.152
SPYM18_RS04860	*topA*	type I DNA topoisomerase	-0.757	2.045
SPYM18_RS04900		CitMHS family transporter	2.246	4.803
SPYM18_RS04905		hypothetical protein	1.719	2.613
SPYM18_RS04910		acetyl-CoA carboxylase biotin carboxyl carrier protein subunit	1.886	3.146
SPYM18_RS04915		sodium ion-translocating decarboxylase subunit beta	2.422	4.054
SPYM18_RS04925	*citD*	citrate lyase acyl carrier protein	3.177	3.108
SPYM18_RS04930	*citE*	citrate (pro-3S)-lyase subunit beta	2.844	3.539
SPYM18_RS04935	*citF*	citrate lyase subunit alpha	3.111	2.788
SPYM18_RS04940	*citX*	citrate lyase holo-[acyl-carrier protein] synthase	3.087	2.914
SPYM18_RS04945		oxaloacetate decarboxylase subunit alpha	2.925	4.536
SPYM18_RS05240		Asp23/Gls24 family envelope stress response protein	1.218	2.118
SPYM18_RS05285	*cfa*	CAMP factor pore-forming toxin Cfa	1.408	2.139
SPYM18_RS05600		helicase-related protein	1.559	2.806
SPYM18_RS05660		helix-turn-helix transcriptional regulator	1.417	2.280
SPYM18_RS05710		sugar ABC transporter permease	-1.656	2.185
SPYM18_RS05775		PTS transporter subunit EIIC	3.118	4.598
SPYM18_RS05780		DUF3284 domain-containing protein	3.401	3.122
SPYM18_RS05785		PTS lactose/cellobiose transporter subunit IIA	3.407	3.334
SPYM18_RS05790		PTS sugar transporter subunit IIB	3.762	2.800
SPYM18_RS05795		PRD domain-containing protein	3.484	4.161
SPYM18_RS05800		DUF871 domain-containing protein	2.878	3.701
SPYM18_RS05805		glycoside hydrolase family 1 protein	2.371	4.862
SPYM18_RS05990	*ptsP*	phosphoenolpyruvate--protein phosphotransferase	-0.850	2.184
SPYM18_RS06210		heavy metal translocating P-type ATPase	-1.034	2.477
SPYM18_RS06215		hypothetical protein	3.224	2.699
SPYM18_RS06220	*sda3*	streptodornase Sda3	1.075	2.364
SPYM18_RS06240		phage holin	2.292	2.404
SPYM18_RS06275		hypothetical protein	1.388	2.046
SPYM18_RS06295		DUF5361 domain-containing protein	1.586	2.560
SPYM18_RS07280		MIP/aquaporin family protein	1.521	2.038
SPYM18_RS07285	*glpO*	type 1 glycerol-3-phosphate oxidase	1.584	3.290
SPYM18_RS07290	*glpK*	glycerol kinase GlpK	1.376	2.409

SPYM18_RS07380		PTS transporter subunit IIC	1.635	2.757
SPYM18_RS07390		PTS sugar transporter subunit IIA	1.077	2.094
SPYM18_RS07510	*sda*	streptodornase A	1.614	2.202
SPYM18_RS07600		minor capsid protein	-3.304	2.031
SPYM18_RS07755		ATP-binding protein	-1.986	2.119
SPYM18_RS07795		helix-turn-helix transcriptional regulator	-2.706	2.286
SPYM18_RS07820	*manL*	PTS sugar transporter subunit IIB	-8.722	5.836
SPYM18_RS07825	*manM*	PTS mannose/fructose/sorbose transporter subunit IIC	-13.64	8.774
SPYM18_RS07830	*manN*	PTS system mannose/fructose/sorbose family transporter subunit IID	-14.11	10.49
SPYM18_RS08040		ABC transporter ATP-binding protein/permease	1.798	4.731
SPYM18_RS08045		ABC transporter ATP-binding protein	1.659	3.604
SPYM18_RS08050		iron ABC transporter permease	1.611	3.447
SPYM18_RS08270		IS30-like element IS1239 family transposase	-1.830	2.457
SPYM18_RS08275		MFS transporter	-2.336	4.238
SPYM18_RS08690		transaldolase	4.373	7.552
SPYM18_RS08695		PTS ascorbate transporter subunit IIC	3.898	6.383
SPYM18_RS08700		PTS sugar transporter subunit IIB	4.827	4.405
SPYM18_RS08705		BglG family transcription antiterminator	4.715	7.972
SPYM18_RS09000	*prsA*	peptidylprolyl isomerase PrsA	-1.712	2.094
SPYM18_RS09160	*hutI*	imidazolonepropionase	2.892	5.045
SPYM18_RS09165		urocanate hydratase	2.735	4.624
SPYM18_RS09170	*ftcD*	glutamate formimidoyltransferase	3.474	4.078
SPYM18_RS09175		cyclodeaminase/cyclohydrolase family protein	3.669	3.624
SPYM18_RS09180		formate--tetrahydrofolate ligase	3.171	3.665
SPYM18_RS09185		HutD family protein	3.688	3.321
SPYM18_RS09190		APC family permease	3.467	3.0688
SPYM18_RS09195	*hutH*	histidine ammonia-lyase	3.642	3.255
SPYM18_RS09205		AAA family ATPase	1.847	5.101
SPYM18_RS09225	*treC*	alpha,alpha-phosphotrehalase	-2.852	3.972
SPYM18_RS09230	*treP*	PTS system trehalose-specific EIIBC component	-2.178	3.308
SPYM18_RS09245		VOC family protein	-1.049	2.052
SPYM18_RS09340	*argR*	arginine repressor	1.213	2.493
SPYM18_RS09445		PadR family transcriptional regulator	-3.553	4.353
SPYM18_RS09450		DUF1700 domain-containing protein	-3.091	5.581
SPYM18_RS09455		DUF4097 family beta strand repeat-containing protein	-3.462	5.016
SPYM18_RS09560	*hasA*	hyaluronan synthase HasA	-1.122	2.193
SPYM18_RS10025	*isdE*	heme ABC transporter substrate-binding protein IsdE	1.540	2.056
SPYM18_RS10325		hypothetical protein	9.701	3.317
SPYM18_RS10340		hypothetical protein	3.110	3.853
SPYM18_RS10620		hypothetical protein	1.468	2.064
SPYM18_RS10635		OadG-related small transporter subunit	3.240	2.656

Note: Only genes with a *p* value above 0.01 are shown. Sequencing available at NCBI Bioproject PRJNA1176168.

Table S5. Differential expression analyses of *S. pyogenes* MGAS82323 Δ*manLMN* and wildtype *S. pyogenes* MGAS8232 at late exponential phase in C-media

Locustag	Gene	Description	$\log_2$ (fold change)	$\log_{10}$ (p value)
SPYM18_RS00275	*purF*	amidophosphoribosyltransferase	1.156	2.525
SPYM18_RS00695	*proC*	pyrroline-5-carboxylate reductase	-1.159	3.204
SPYM18_RS00700	*pepA*	glutamyl aminopeptidase	-1.165	2.425
SPYM18_RS00900	*nga*	nicotine adenine dinucleotide glycohydrolase	1.448	2.223
SPYM18_RS01035		transglutaminase domain-containing protein	1.546	2.455
SPYM18_RS01075		MarR family transcriptional regulator	0.976	2.278
SPYM18_RS01135	*rnpA*	ribonuclease P protein component	-0.901	2.420
SPYM18_RS01160		ABC transporter substrate-binding protein	-1.025	2.192
SPYM18_RS01200		TatD family hydrolase	-0.963	2.325
SPYM18_RS01430		class I SAM-dependent methyltransferase	1.174	2.363
SPYM18_RS01705		phage portal protein	-1.791	3.496
SPYM18_RS01715		phage major capsid protein	-1.859	3.848
SPYM18_RS01725		hypothetical protein	-1.940	2.174
SPYM18_RS01735		hypothetical protein	-1.252	2.012
SPYM18_RS01745		hypothetical protein	-1.342	2.736
SPYM18_RS01755		phage tail tape measure protein	-1.372	2.795
SPYM18_RS01760		phage tail family protein	-1.769	2.378
SPYM18_RS01765		phage tail spike protein	-1.578	2.716
SPYM18_RS01770		hyaluronoglucosaminidase	-2.031	2.678
SPYM18_RS01875	*racE*	glutamate racemase	0.905	2.336
SPYM18_RS02090	*nrdI*	class Ib ribonucleoside-diphosphate reductase assembly flavoprotein NrdI	1.374	2.093
SPYM18_RS02185		peptidylprolyl isomerase	-1.783	2.531
SPYM18_RS02325		IS1182 family transposase	-1.291	2.765
SPYM18_RS02460		VOC family protein	-0.804	2.064
SPYM18_RS02750		PRD domain-containing protein	1.298	2.125
SPYM18_RS02785		SprT family protein	-1.310	2.004
SPYM18_RS02885	*pepF*	oligoendopeptidase F	-0.941	2.079
SPYM18_RS02930		HD domain-containing protein	-1.563	3.942
SPYM18_RS03005	*queG*	tRNA epoxyqueuosine(34) reductase QueG	-1.121	2.985
SPYM18_RS03055		YvcK family protein	-1.616	2.674
SPYM18_RS03090		phage repressor protein/antirepressor Ant	2.863	3.835
SPYM18_RS03130		recombinase RecT	2.003	2.524
SPYM18_RS03140		PD-(D/E)XK nuclease-like domain-containing protein	2.830	3.035
SPYM18_RS03150		hypothetical protein	2.459	2.922
SPYM18_RS03170		YopX family protein	3.485	2.626
SPYM18_RS03180		class I SAM-dependent methyltransferase	2.368	2.585
SPYM18_RS03185		site-specific DNA-methyltransferase	2.750	3.197
SPYM18_RS03190		DUF1642 domain-containing protein	2.595	2.750
SPYM18_RS03200		DUF1492 domain-containing protein	1.829	3.626

SPYM18_RS03235		terminase large subunit	1.346	2.859
SPYM18_RS03240		hypothetical protein	2.747	2.195
SPYM18_RS03245		phage portal protein	1.366	2.586
SPYM18_RS03250		Clp protease ClpP	1.615	2.074
SPYM18_RS03260		head-tail connector protein	1.567	2.228
SPYM18_RS03270		HK97 gp10 family phage protein	1.478	2.071
SPYM18_RS03295		phage tail tape measure protein	1.372	2.143
SPYM18_RS03325		DUF1366 domain-containing protein	3.780	2.216
SPYM18_RS03365		zinc ABC transporter substrate-binding protein AdcA	1.176	2.305
SPYM18_RS03655		glycosyltransferase family 2 protein	1.191	2.531
SPYM18_RS03675		rhamnan synthesis F family protein	3.621	2.525
SPYM18_RS03705	*pepT*	peptidase T	1.001	2.501
SPYM18_RS03775		membrane protein	-1.777	3.303
SPYM18_RS03885		leucine-rich repeat domain-containing protein	1.383	3.2470
SPYM18_RS04045		hydroxymethylglutaryl-CoA reductase, degradative	-1.374	3.372
SPYM18_RS04085		ATP-dependent Clp protease ATP-binding subunit	1.883	2.312
SPYM18_RS04135	*pyrE*	orotate phosphoribosyltransferase	1.257	2.453
SPYM18_RS04425	*hemW*	radical SAM family heme chaperone HemW	-1.01	2.600
SPYM18_RS04615		2-hydroxycarboxylate transporter family protein	-2.260	3.584
SPYM18_RS04620		NADP-dependent malic enzyme	-1.318	2.418
SPYM18_RS04695		RluA family pseudouridine synthase	0.900	2.014
SPYM18_RS04765		L-threonylcarbamoyladenylate synthase	-1.254	2.105
SPYM18_RS04770		GNAT family N-acetyltransferase	-1.327	3.259
SPYM18_RS04775		serine hydroxymethyltransferase	-1.365	2.358
SPYM18_RS04790		ABC transporter ATP-binding protein	-1.184	2.016
SPYM18_RS04900		CitMHS family transporter	1.316	2.612
SPYM18_RS04905		hypothetical protein	-1.646	2.344
SPYM18_RS04990	*guaA*	glutamine-hydrolyzing GMP synthase	-1.008	2.089401
SPYM18_RS04995		lipid II:glycine glycyltransferase FemX	1.481	2.648
SPYM18_RS05000		ABC-F family ATP-binding cassette domain-containing protein	1.505	3.188
SPYM18_RS05070	*coaB*	phosphopantothenate--cysteine ligase	-1.249	2.646
SPYM18_RS05100		ABC transporter ATP-binding protein	1.066	2.256
SPYM18_RS05200	*truB*	tRNA pseudouridine(55) synthase TruB	-1.113	2.226
SPYM18_RS05260	*amaP*	alkaline shock response membrane anchor protein AmaP	1.367	2.015
SPYM18_RS05480		major capsid protein	1.086	2.3569
SPYM18_RS05490		DUF4355 domain-containing protein	1.515	2.582
SPYM18_RS05520		phage head morphogenesis protein	-1.549	2.232
SPYM18_RS05585		bifunctional DNA primase/polymerase	2.043	2.685
SPYM18_RS05590		hypothetical protein	1.585	2.725
SPYM18_RS05595		DUF669 domain-containing protein	2.729	3.263
SPYM18_RS05600		helicase-related protein	2.064	3.839
SPYM18_RS05605		AAA family ATPase	1.889	2.115
SPYM18_RS05620		hypothetical protein	2.955	3.825

SPYM18_RS05630		helix-turn-helix transcriptional regulator	1.545	2.273
SPYM18_RS05665		DUF4145 domain-containing protein	-2.077	2.493
SPYM18_RS05675		type II toxin-antitoxin system PemK/MazF family toxin	-0.844	2.067
SPYM18_RS05720		IS30 family transposase	1.150	2.074
SPYM18_RS06085		pseudouridine synthase	1.419	2.19
SPYM18_RS06115	*sodA*	superoxide dismutase SodA	-1.865	2.854
SPYM18_RS06125		DNA internalization-related competence protein ComEC/Rec2	0.913	2.158
SPYM18_RS06220	*sda3*	streptodornase Sda3	-2.216	4.883
SPYM18_RS06275		hypothetical protein	1.468	2.230
SPYM18_RS06290		hypothetical protein	1.432	2.025
SPYM18_RS06365		hypothetical protein	0.866	2.003
SPYM18_RS06415		hypothetical protein	1.599	2.050
SPYM18_RS06425		PD-(D/E)XK nuclease-like domain-containing protein	1.739	2.228
SPYM18_RS06430		recombinase RecT	1.956	2.038
SPYM18_RS06650	*typA*	translational GTPase TypA	-0.971	2.303
SPYM18_RS06670		Dps family protein	-1.291	2.530
SPYM18_RS06735		GNAT family N-acetyltransferase	-1.277	2.922
SPYM18_RS06750		arginine repressor	-1.030	2.733
SPYM18_RS06770		response regulator	-1.094	2.667
SPYM18_RS06920		glycoside hydrolase family 125 protein	-1.489	2.192
SPYM18_RS06925		alpha-mannosidase	-1.255	2.423
SPYM18_RS07035		S1 RNA-binding domain-containing protein	-1.168	2.387
SPYM18_RS07130		S-ribosylhomocysteine lyase	4.833	2.497
SPYM18_RS07155	*recU*	Holliday junction resolvase RecU	-0.905	2.283
SPYM18_RS07225	*rsmH*	16S rRNA (cytosine(1402)-N(4))-methyltransferase RsmH	-1.533	2.482
SPYM18_RS07270		helix-turn-helix domain-containing protein	-0.936	2.071
SPYM18_RS07275		FAD/NAD(P)-binding oxidoreductase	-1.090	2.214
SPYM18_RS07480		hypothetical protein	-2.102	3.825
SPYM18_RS07490		N-acetyltransferase	1.436	2.487
SPYM18_RS07575		tape measure protein	-2.875	2.538
SPYM18_RS07590		hypothetical protein	-2.360	2.532
SPYM18_RS07620		hypothetical protein	-2.460	2.799
SPYM18_RS07645		PBSX family phage terminase large subunit	-1.275	2.479
SPYM18_RS07785		phage antirepressor KilAC domain-containing protein	-2.264	2.767
SPYM18_RS07805		DUF4041 domain-containing protein	-5.11	3.15
SPYM18_RS07810		site-specific integrase	2.864	3.268
SPYM18_RS07820	*manL*	PTS sugar transporter subunit IIB	-12.13	6.654
SPYM18_RS07825	*manM*	PTS mannose/fructose/sorbose transporter subunit IIC	-14.38	8.951
SPYM18_RS07830	*manN*	PTS system mannose/fructose/sorbose family transporter subunit IID	-12.81	10.01
SPYM18_RS07850	*accD*	acetyl-CoA carboxylase, carboxyltransferase subunit beta	0.950	2.057
SPYM18_RS07860	*fabZ*	3-hydroxyacyl-ACP dehydratase FabZ	-3.614	2.241

SPYM18_RS07880	*fabD*	ACP S-malonyltransferase	1.453	2.484
SPYM18_RS07915	*dnaK*	molecular chaperone DnaK	1.753	2.134
SPYM18_RS07920	*grpE*	nucleotide exchange factor GrpE	2.098	2.308
SPYM18_RS07925	*hrcA*	heat-inducible transcriptional repressor HrcA	1.389	2.973
SPYM18_RS07990		universal stress protein	-1.278	2.113
SPYM18_RS08010		ABC transporter ATP-binding protein	1.773	3.145
SPYM18_RS08040		ABC transporter ATP-binding protein/permease	1.563	4.144
SPYM18_RS08045		ABC transporter ATP-binding protein	1.209	2.460
SPYM18_RS08050		iron ABC transporter permease	1.228	2.450
SPYM18_RS08070		CHAP domain-containing protein	1.099	2.171
SPYM18_RS08155		magnesium transporter CorA family protein	-1.086	2.293
SPYM18_RS08325	*deoC*	deoxyribose-phosphate aldolase	1.426	2.600
SPYM18_RS08360	*tsaB*	tRNA (adenosine(37)-N6)-threonylcarbamoyltransferase complex dimerization subunit type 1 TsaB	-1.267	2.555
SPYM18_RS08365		DNA-dependent RNA polymerase subunit epsilon	-1.582	2.976
SPYM18_RS08565	*lacG*	6-phospho-beta-galactosidase	1.838	2.172
SPYM18_RS08575		PTS lactose/cellobiose transporter subunit IIA	2.202	2.362
SPYM18_RS08585		tagatose-6-phosphate kinase	1.649	2.397
SPYM18_RS08590	*lacB*	galactose-6-phosphate isomerase subunit LacB	3.308	2.668
SPYM18_RS08595	*lacA*	galactose-6-phosphate isomerase subunit LacA	1.211	2.031
SPYM18_RS08690		transaldolase	-1.657	3.326
SPYM18_RS08695		PTS ascorbate transporter subunit IIC	-1.227	2.061
SPYM18_RS08705		BglG family transcription antiterminator	-1.347	2.766
SPYM18_RS08935	*scpA/B*	C5a peptidase ScpA/B	1.312	2.478
SPYM18_RS09130		ATP-dependent Clp protease ATP-binding subunit	0.866	2.084
SPYM18_RS09135		CtsR family transcriptional regulator	1.134	2.812
SPYM18_RS09150	*ahpC*	alkyl hydroperoxide reductase subunit C	-1.420	2.427
SPYM18_RS09255	*nrdG*	anaerobic ribonucleoside-triphosphate reductase activating protein	1.335	2.483
SPYM18_RS09420		cell division protein FtsK	1.834	2.183
SPYM18_RS09440		CoA pyrophosphatase	1.121	2.404
SPYM18_RS09495	*mnmG*	tRNA uridine-5-carboxymethylaminomethyl(34) synthesis enzyme MnmG	0.996	2.489
SPYM18_RS09525		energy-coupling factor transporter transmembrane protein EcfT	1.150	2.177
SPYM18_RS09545		helix-turn-helix domain-containing protein	1.054	2.367
SPYM18_RS09560	*hasA*	hyaluronan synthase HasA	1.112	2.235
SPYM18_RS09820		IS3 family transposase	-2.146	3.17
SPYM18_RS09880		DUF3042 family protein	-1.669	2.335
SPYM18_RS10005		GNAT family N-acetyltransferase	-1.952	2.091
SPYM18_RS10280		SPJ_0845 family protein	-1.812	2.110
SPYM18_RS10295		hypothetical protein	2.256	2.180
SPYM18_RS10305		hypothetical protein	1.986	2.434
SPYM18_RS10440		hypothetical protein	-3.310	2.698
SPYM18_RS10575		8-oxo-dGTP diphosphatase	-0.841	2.143

SPYM18_RS10620	hypothetical protein	1.831	2.783
SPYM18_RS10640	DEAD/DEAH box helicase family protein	2.050	2.577

Note: Only genes with a *p* value above 0.01 are shown. Sequencing is available at NCBI Bioproject PRJNA1176168.

Table S6. RNA sequencing reads for all samples

Sample	Total reads	All mapped reads	CDS mapped reads
**WT1_THY**	27154336	27098496	10887685
**WT2_THY**	21387960	21328611	17555484
**WT3_THY**	25554376	25094650	20078396
**Manlmn1_THY**	40834088	40769557	15984286
**Manlmn2_THY**	31004436	30642800	24317558
**Manlmn3_THY**	30431000	30157963	25468920
**WT1_Cmed**	13811616	13085719	6960313
**WT2_Cmed**	18713826	18556043	11114139
**WT3_Cmed**	25427390	24636518	16739946
**Manlmn1_Cmed**	24973278	24523166	12621209
**Manlmn2_Cmed**	22085292	21814211	12145513
**Manlmn3_Cmed**	22140958	21529626	14916243

Note: Sequencing data has been deposited at NCBI Bioproject PRJNA1176168. CDS – coding sequence
